# Deregulation of SOCS5 suppresses dendritic cell function in chronic lymphocytic leukemia

**DOI:** 10.18632/oncotarget.10093

**Published:** 2016-06-15

**Authors:** Patricia A. Toniolo, Suhu Liu, Jennifer E. Yeh, Darwin Q. Ye, José Alexandre M. Barbuto, David A. Frank

**Affiliations:** ^1^ Department of Medical Oncology, Dana-Farber Cancer Institute, and Department of Medicine, Brigham and Women's Hospital and Harvard Medical School, Boston, MA, USA; ^2^ Department of Immunology, Institute of Biomedical Sciences, University of Sao Paulo, Sao Paulo, Brazil

**Keywords:** monocytes, dendritic cells, SOCS5, CLL, STAT3

## Abstract

One cause of morbidity and mortality in chronic lymphocytic leukemia (CLL) is infection, which results from defects in a number of components of the immune system. In particular, dendritic cells (DCs) are functionally defective in patients with CLL. To understand the molecular mechanism for this abnormality, we focused on signal transduction pathways that regulate the function of monocyte-derived dendritic cells (Mo-DCs). Monocytes from CLL patients exhibit high IL-4Rα expression due to the enhanced activation of STAT3. However, IL-4R signaling is decoupled from activation of its downstream mediator STAT6 by enhanced levels of the negative regulator SOCS5. This impairs differentiation of functionally mature DCs leading to decreased expression of HLA-DR and costimulatory molecules, and reduced secretion of pro-inflammatory cytokines in LPS-activated DCs. Moreover, Mo-DCs from CLL patients display a decreased ability to induce pro-inflammatory T-cell responses. IL-10-treatment of monocytes from healthy donors mimics the alteration in signaling observed in CLL patients, through enhanced STAT3-dependent expression of SOCS5. The higher level of SOCS5 inhibits STAT6 activation and leads to defective DC differentiation. These findings indicate that SOCS5 mediates the impaired function of DCs in CLL patients, and has the potential to be a new therapeutic target for reversing cancer-associated immune suppression.

## INTRODUCTION

Chronic lymphocytic leukemia (CLL) is a malignancy of monoclonal mature B cells that represents the most common form of adult leukemia in Western countries. These malignant B-cells accumulate in the peripheral blood and in the primary and secondary lymphoid organs [1, 2]. Although the clinical course of CLL is highly variable [3, 4], dysfunction of several other immune cells occurs at early disease stages [5–9] leading to increased susceptibility to infections, a common complication and major cause of morbidity and mortality [10, 11].

Dendritic cells (DCs), which are sentinel cells of the immune system and the most important antigen-presenting cells, are required to generate potent anti-tumor immunity [12, 13]. They support the effector function of important downstream immune cells critical to fight both infection and neoplasia, such as natural killer, B and T cells [13, 14]. However, immunosuppressive factors released by malignant cells can affect DC function. Although it is known that DCs from CLL patients are defective in antigen presentation [5, 15], the molecular mechanism driving these alterations in DCs is still unclear. Given the key function of DCs in the immune response, a better understanding of their physiology as well as strategies to overcome tumor-induced DC dysfunction has the potential to improve cancer treatment.

Several cytokines and grown factors important for both DC differentiation and tumor progression activate transcription factors of the signal transducer and activator of transcription (STAT) family [16, 17]. STAT6 is activated following IL-4 stimulation, and, together with GM-CSF-induced STAT5, leads to differentiation of DCs from circulating monocytes [18–20]. Inhibition of the STAT6 pathway during differentiation of monocyte-derived DC (Mo-DC) prevents the acquisition of a mature phenotype by these DCs [20, 21]. In addition, factors released by malignant cells like IL-10 and IL-6 induce activation of STAT3, and this results in abnormal DC differentiation [15, 22, 23].

Given that STATs regulate genes controlling key cellular processes, the activation state of STATs is tightly regulated. The magnitude and duration of STAT activation are negatively regulated by an 8-member family of suppressor of cytokine signaling (SOCS) proteins [16]. Among SOCS proteins, SOCS5 regulates the activation of both STAT6 and STAT3 [24–26]. SOCS5 has also been shown to be downregulated in cancer cells, and may play a role in reducing tumor growth and angiogenesis [25, 27].

Herein, we explored the role of SOCS5 and STAT signaling in the differentiation of Mo-DCs from patients with CLL, as a way to both understand the immune deficiency in this disease and develop approaches for targeted therapy.

## RESULTS

### Mo-DCs from CLL patients display an impaired phenotype

**Figure 1:**
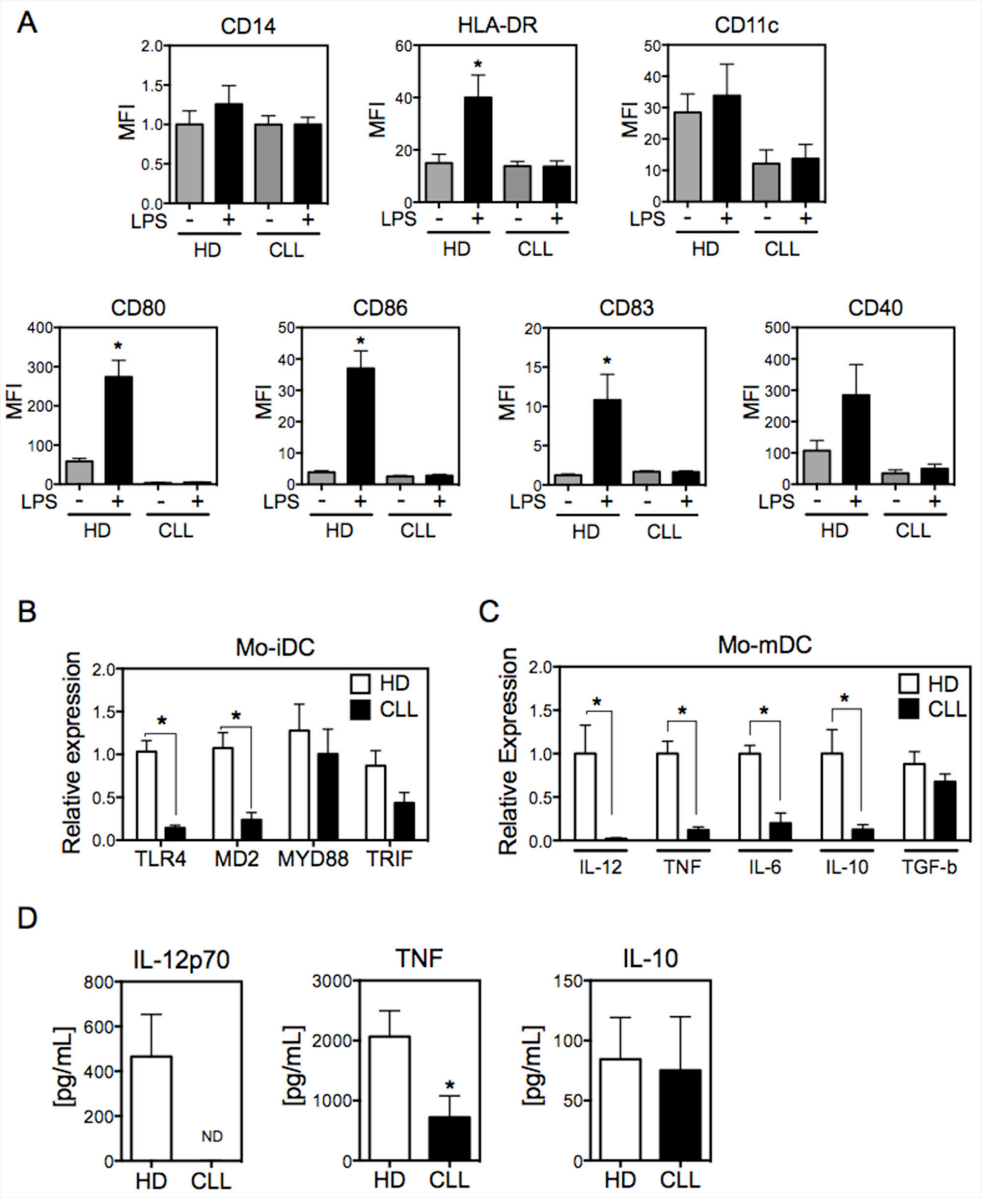
Mo-DCs derived from CLL patients display impaired maturation **A.** Mo-iDCs (in the absence of LPS) and Mo-mDCs (LPS-stimulated for 24h) were differentiated from monocytes derived from CLL patients and healthy donors, and were analyzed for the expression of DC surface markers by flow cytometry. Doublets were excluded from analysis and Mo-DCs were defined as CD14^−^HLA-DR^+^. N=6. One-way ANOVA Tukey multiple comparison test. *p<0.05, compared with other groups. **B.** RNA from Mo-iDCs was analyzed by qRT-PCR for the expression of the indicated genes, normalized to 18S RNA. N=5. Unpaired t-test. *p<0.05. **C.** The expression by Mo-mDCs of the indicated cytokines was analyzed by qRT-PCR, normalized to 18S RNA. N=8. Unpaired t-test. *p<0.05. **D.** Cytokine production was measured by ELISA from the supernatant of Mo-mDCs from CLL patients and healthy donors. N=12. Unpaired t-test. *p<0.05. Data are shown as means ± SEM.

To better understand the molecular underpinnings for DC dysfunction in CLL, we first evaluated the expression of surface markers of Mo-DCs derived from patients with this disease. Mo-DCs were differentiated in the presence of IL-4 and GM-CSF for five days and stimulated with LPS for 24 hours to obtain mature Mo-DCs (Mo-mDCs). As expected, Mo-DCs from healthy donors displayed a mature phenotype following LPS stimulus, reflected by the high expression of HLA-DR, CD80, CD86, CD83, and CD40, while maintaining elevated expression of CD11c (Figure 1A). However, Mo-DCs from CLL patients showed poor maturation after LPS stimulation. There was essentially no induction of expression of HLA-DR, the costimulatory molecules CD80 and CD86, CD83 or CD40. Also, Mo-iDCs from CLL patients showed lower levels of CD11c expression than Mo-iDCs from healthy donors, with little change after LPS stimulation. Although the expression of CD80 and CD40 were considerably higher in CLL Mo-mDCs than in CLL Mo-iDCs (Supplementary Figure S1A), this enhancement was insignificant when compared with Mo-DCs from healthy donors (Figure 1A). The activation of DCs by LPS requires TLR4, CD14, MD2 and the signaling molecules MyD88 and TRIF [28]. Interestingly, immature Mo-DCs from CLL patients expressed lower mRNA levels of TLR4 and its associated protein MD2, with a trend toward reduced expression of MyD88 and TRIF, when compared with healthy donors (Figure 1B). Therefore, the impaired maturation of CLL Mo-DCs after LPS stimulation may be related to the defective expression of TLR4 and its associated molecules. In the presence of elevated LPS concentrations (500 ng/mL) CLL Mo-DC showed increased expression of surface molecules when compared with lower dose of LPS (50 ng/mL) (Supplementary Figure S1B). However, even this enhancement was not comparable to the levels of Mo-DCs from healthy donors, which indicates that patients' Mo-DCs are less responsive to LPS. To determine whether this effect is restricted to the TLR4 pathway, we evaluated mRNA levels of TLR3, TLR5, TLR7 and TLR9 and the expression of receptors for TNF, PGE2, IL-6 and IL-1β. Similar to TLR4, other TLRs were downregulated in CLL Mo-iDCs (Supplementary Figure S1C). Also, the TNF receptor CD120a, a high affinity receptor that mediates DCs maturation, [29] was reduced in CLL Mo-iDCs, as were the receptors for IL-6 and IL-1 β (Supplementary Figure S1D). These findings indicate that Mo-DCs from CLL patients are not activated efficiently in the presence of a broad spectrum of stimuli, reflecting an overall deficiency of Mo-DC maturation in these patients.

To further evaluate the phenotype of Mo-DCs derived from CLL patients, we next explored the pattern of pro-inflammatory and anti-inflammatory cytokines produced by these cells compared to normal Mo-DCs. As expected, LPS-activated Mo-DCs from normal donors expressed the pro-inflammatory cytokines IL-12, TNF and IL-6, as well as the anti-inflammatory cytokine IL-10 and the immunosuppressive cytokine TGF-β. However, CLL-derived Mo-DCs showed reduced expression of pro-inflammatory cytokines and IL-10, while expressing TGF-β at similar levels to that of normal donors (Figure 1C). Consistent with this phenotype, large amounts of IL12p70, TNF and IL-10 were released by LPS-activated Mo-DCs from normal donors, whereas only IL-10 and low levels of TNF were detected in the supernatant of CLL Mo-DCs (Figure 1D). Together, these results confirm that CLL Mo-DCs have an impaired phenotype, reflecting the inability to fully differentiate to mature Mo-DCs.

### CLL Mo-DC induces reduced allogeneic T-cell response

**Figure 2:**
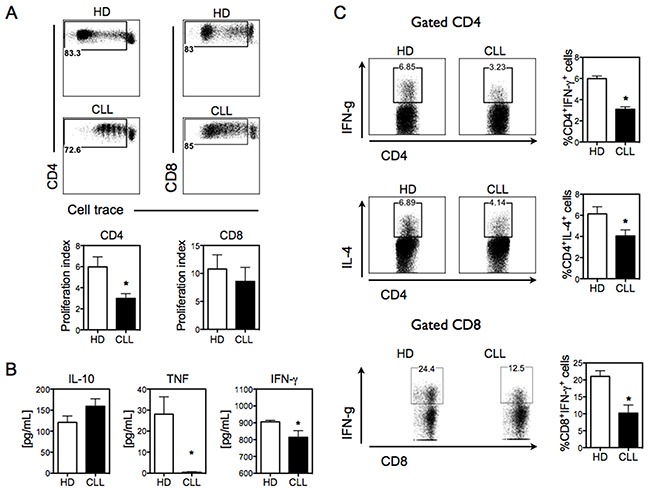
CLL Mo-DCs shows deficient induction of pro-inflammatory allogeneic T-cell response **A.** Mo-mDCs from healthy donors (HD) and CLL patients (CLL) were co-cultured with bead-purified allogeneic CD3 T-cells for 5 days, and T-cell proliferation was measured by CellTrace dilution assessed by flow cytometry. Plots in the upper panel are representative of CD4+ and CD8+ T-cell proliferation from a representative experiment. Numbers in the plots represent the percentage of proliferating cells. The proliferation index of CD4^+^ and CD8^+^ T cells was calculated with FlowJo 8.7 software (*bottom panel*). N=7 run in duplicate or triplicate. **B.** Cytokine secretion in the supernatant of co-cultures of CD3^+^ T-cells and Mo-mDCs was measured by ELISA. N=14 for IL-10 and N=8 for the other cytokines. **C.** CD3^+^ T cells co-cultured with Mo-mDCs were analyzed by intracellular cytokine staining. *Upper plots* show IFNγ-producing CD4^+^ T cells. *Middle plots* show IL-4-producing CD4^+^ T cells. *Bottom plots* show IFNγ-producing CD8^+^ T cells. Graphical representation of the frequency of CD4^+^IFNγ^+^, CD4^+^ IL-4^+^ and CD8^+^ IFNγ^+^ is shown on the right of the respective plots. N=3 run in triplicate. Data are shown as mean ± SEM. Unpaired t-test. *p<0.05.

Having shown that Mo-DCs derived from CLL patients with active disease have a defective phenotype, we next determined the ability of CLL Mo-DCs to induce allogeneic T-cell response *in vitro*. Allogeneic CD3^+^ T cells were cultured with LPS-activated Mo-DCs derived from healthy donors or CLL patients, and the proliferation of T-cells was analyzed through cell trace dilution with flow cytometry. While normal Mo-DCs were able to stimulate robust proliferation of both CD4^+^ and CD8^+^ T cells, CLL Mo-DCs demonstrated a lower CD4^+^ T-cells allostimulatory capacity and did not significantly stimulate CD8^+^ T-cell proliferation (Figure 2A).

We next assessed whether CLL Mo-DCs could affect the pattern of T-cell-mediated response by measuring the cytokines produced in the co-culture supernatant. Mo-DCs from healthy donors led to the secretion of pro-inflammatory (IFN-γ and TNF) and anti-inflammatory (IL-10) cytokines by CD3^+^ T cells. In contrast, only IL-10 and low levels of IFN-γ were released when these lymphocytes were stimulated by CLL Mo-DCs (Figure 2B). Interestingly, while Mo-DCs from some CLL patients induced CD8^+^ T-cell proliferation comparable to Mo-DCs from healthy donors, the frequency of IFN-γ -producing CD8^+^ T-cells was strongly reduced (Figure 2C). These data show that Mo-DCs from CLL patients have a low capacity to induce pro-inflammatory cytokines by T cells.

To further explore the ability of CLL Mo-DCs to alter the polarization of CD4^+^ T-cells toward Th1 or Th2 differentiation, we determined the percentage of IFN-γ (Th1-type)- and IL-4 (Th2-type)-producing cells by intracellular cytokine staining. While Mo-DCs from healthy donors led to strong induction of IFN-γ- and IL-4-producing T-cells, CLL Mo-DCs showed greatly diminished activity (Figure 2C). These findings indicate that CLL Mo-DCs have lower capacity to induce T-cell polarization.

Previous reports have demonstrated increased numbers of Tregs in patients with CLL, which may contribute to the diminished immune response in these patients. [7, 30] Thus, we next determined whether CLL Mo-DCs have the potential to induce and expand Tregs. Consistent with the immunosuppressive phenotype of CLL Mo-DCs, a higher frequency of Tregs (CD4^+^CD127^lo^CD25^hi^FOXP3^+^) was induced from co-culture with CLL Mo-DCs compared to co-culture with normal Mo-DCs (Supplementary Figure S2). Taken together, these data confirm functional impairment of Mo-DCs from CLL.

### IL-4α receptor is upregulated in monocytes from CLL

**Figure 3:**
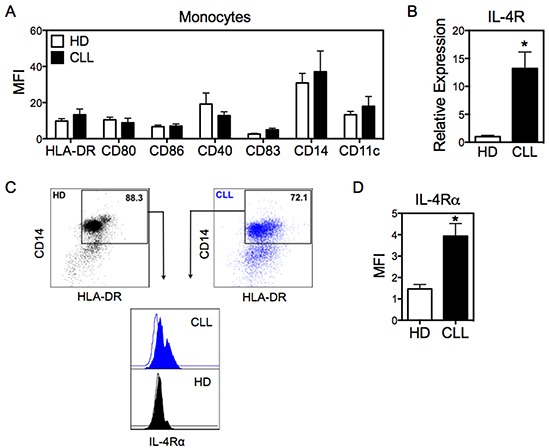
Monocytes from CLL patients exhibit high expression of IL-4Rα **A.** Monocytes from CLL and HD were analyzed by flow cytometry for expression of the indicated surface markers. The monocyte population was defined as CD14^+^HLA-DR^+^, after doublet exclusion. N=7, HD. N=9, CLL. **B.** mRNA expression of IL-4Rα in monocytes was evaluated by qRT-PCR, normalized to 18S RNA. N=5. **C.** Expression of IL-4Rα was determined by flow cytometry in monocytes from CLL patients and HD. Analysis was performed in the population of cells defined as in panel (A). **D.** IL-4Rα expression was determined by flow cytometry in monocytes from CLL patients and HD. N=5. Data are shown as mean ± SEM. Unpaired t-test. *p<0.05.

Given that Mo-DCs from CLL patients have impaired production of pro-inflammatory cytokines and cell surface markers associated with maturation status, we considered the possibility that monocytes from these patients displayed molecular changes that impaired their full differentiation into DCs. First, we analyzed monocytes from CLL patients for expression of the surface makers CD14, CD11c, HLA-DR, CD80, CD86, CD40, and CD83 by flow cytometry. The expression of these molecules was similar between monocytes from CLL patients and healthy donors (Figure 3A). Therefore, we next evaluated the expression of IL-4 receptor alpha (IL-4Rα) in monocytes, since IL-4 is required for the *in vitro* differentiation in DCs [19]. Interestingly, IL-4Rα was prominently enhanced in monocytes from CLL patients compared to healthy donors, both at the mRNA level (Figure 3B) and at the protein level (Figure 3C and 3D).

### Impaired STAT6 phosphorylation in monocytes from CLL patients is associated with increased SOCS5 expression

**Figure 4:**
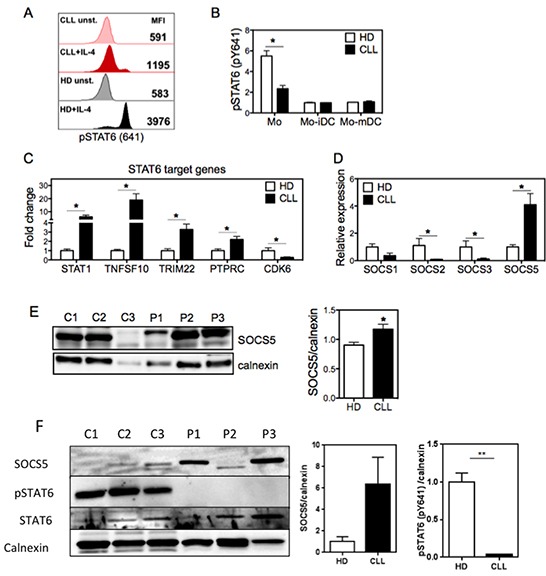
Monocytes from CLL patients show decreased STAT6 phosphorylation in the setting of increased expression of the negative regulator SOCS5 **A.** Monocytes from CLL patients and HD were left unstimulated or stimulated with IL-4 for 15 min, and the phosphorylation of tyrosine-641 of STAT6 (pSTAT6) was assessed by flow cytometry. Representative histograms show median fluorescence intensity (MFI) of pSTAT6 in monocytes, as indicated. **B.** Graphical representation of pSTAT6 in monocytes and Mo-DCs (stimulated as described in (A)). Data are shown as the ratio of pSTAT6 MFI of IL-4-stimulated cells to pSTAT6 MFI of unstimulated cells. For (A) and (B), analysis was performed in the region (FCS x SSC) referent to monocytes or Mo-DCs, excluding doublets. N=4. **C.** mRNA of STAT6 target genes was accessed in monocytes from CLL and HD by qRT-PCR normalized to 18S RNA. Fold increase was determined by dividing the expression in each sample by that of average of HD. N=5. **D.** mRNA expression of the indicated SOCS family members was compared between CLL and HD by qRT-PCR (normalized to 18S RNA). N=5. **E.** Lysates of monocytes from HD controls (C) and CLL patients (P) were analyzed by immunoblotting for SOCS5 (left panel). Intensity of bands was quantified and normalized to the loading control calnexin (right panel). N=3. Data are shown as mean ± SEM, and were analyzed by an unpaired t-test. *p<0.05. **F.** Lysates of monocytes from HD controls (C) and CLL patients (P) (both of which were independent of the samples used in (E)) were analyzed by immunoblotting for SOCS5 and total and phosphorylated STAT6 (left panel). Intensity of indicated bands was quantified and normalized to the loading control calnexin (right panel). N=3. Data are shown as mean ± SEM, and were analyzed by an unpaired t-test. **p<0.01.

Since IL-4 signals through IL-4R via STAT6 [20], we assessed whether STAT6 activity was altered in CLL monocytes. We found that monocytes from CLL patients exhibited prominently lower levels of the activating tyrosine phosphorylation of STAT6 following IL-4 stimulation (Figure 4A). During the differentiation of these monocytes into DCs, IL-4-induced STAT6 phosphorylation decreases, as has been reported from a mouse model [20], and the levels of phosphorylated STAT6 in immature and mature Mo-DCs were similar in cells derived from CLL patients and healthy donors (Figure 4B). These findings indicate that STAT6 signaling, which is critical during early stage of Mo-DC differentiation [21, 31], is strongly attenuated in monocytes from CLL patients. Since IL-4R is highly expressed in CLL monocytes, we considered whether increased concentrations of IL-4 during the differentiation of monocytes into DCs would be sufficient to restore the altered phenotype of CLL Mo-DCs. However, even elevated doses of IL-4 could not restore normal expression of HLA-DR and costimulatory molecules in CLL-derived Mo-DCs (Supplementary Figure S3A). To determine whether the reduced phosphorylation of STAT6 in CLL monocytes affects its transcriptional activity, we evaluated the mRNA expression of endogenous STAT6 target genes [32] (Figure 4C). The expression of *Stat1*, *Tnfsf10*, *Ptprc* and *Trim22*, which are repressed by STAT6, was increased in CLL monocytes, whereas *Cdk6*, which is induced by STAT6, was downregulated. These findings indicate that the reduced phosphorylation of STAT6 results in decreased STAT6 transcriptional activity.

To further understand why STAT6 activity is inhibited in monocytes from CLL patients despite their high levels of IL-4R, we considered the possibility that a negative regulator of this pathway, such as a SOCS protein, was more highly expressed in monocytes in CLL patients. Among the best characterized SOCS family members in Mo-DCs, expression of SOCS1, SOCS2, and SOCS3 were all significantly lower in monocytes from CLL patients compared to healthy controls. By contrast, the expression of SOCS5 was significantly elevated in CLL monocytes at both the mRNA (Figures 4D) and protein level (Figures 4E), compared with healthy monocytes. This increased expression of SOCS5 correlated closely with the decreased phosphorylation of STAT6 detected in the patient samples (Figure 4F). Furthermore, the expression of SOCS5 was independent of IL-4 stimulation (Supplementary Figure S3B). These findings raised the possibility that a deregulation in the expression of SOCS5 in CLL monocytes could be responsible for the defective differentiation of Mo-DCs through inhibiting STAT6 phosphorylation.

### IL-10-induced STAT3 regulates the expression of SOCS5 in monocytes

**Figure 5:**
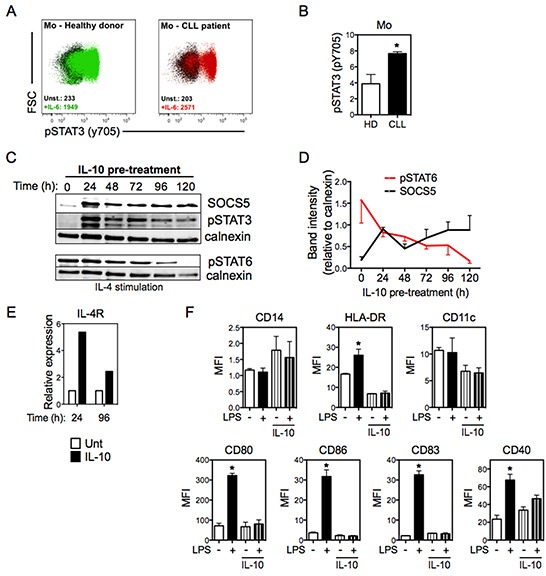
IL-10 induces the expression of both SOCS5 and IL-4Rα **A.** Representative distribution of phosphorylation of tyrosine-705 of STAT3 (pSTAT3) in monocytes from CLL patients and HD, left unstimulated (unst.) or stimulated with IL-6 (+IL-6) for 15 min, as analyzed by flow cytometry. Numbers in the plots show MFI values for pSTAT3. **B.** Quantitation of pSTAT3 in monocytes, stimulated as described in (A). Data are shown as the ratio of pSTAT3 MFI for IL-4-stimulated cells to pSTAT6 MFI for unstimulated cells. N=4. Unpaired t-test. **C.** Monocytes from HD were treated with IL-10 (50 ng/mL) at the indicated time points and immunoblots were performed for SOCS5, pSTAT3 and pSTAT6. For pSTAT6 analyses cells were additionally stimulated with IL-4 for 30 min (bottom panel). Calnexin was used as loading control. **D.** Indicated bands from (C) were quantitated relative to calnexin. N=3. **E.** IL-4Rα mRNA expression in HD monocytes treated and untreated with IL-10 was quantitated by qRT-PCR at the indicated time points (normalized to 18S RNA). **F.** Mo-iDCs (in the absence of LPS) and Mo-mDCs (LPS-stimulated for 24h) were differentiated from HD monocytes in the presence or absence of IL-10 (50 ng/mL), and were analyzed for the expression of DC surface markers by flow cytometry. Doublets were excluded from analysis and Mo-DCs were defined as CD14^−^HLA-DR^+^. N=4. One-way ANOVA Tukey multiple comparison test. *p<0.05, compared with other groups. Data are shown as mean ± SEM.

We next focused on understanding the mechanism for the SOCS5 overexpression seen in CLL-derived monocytes. It is known that patients with CLL display high serum levels of IL-10, which can be released by leukemic B cells and affect DC function [15, 23, 33]. Since IL-10 signals through activation of STAT3, we first evaluated whether phosphorylation of STAT3 is altered in CLL-derived monocytes. Consistent with an effect of IL-10, monocytes from CLL patients exhibited high levels of the activating tyrosine phosphorylation of STAT3 compared to normal monocytes (Figure 5A and 5B). Furthermore, analysis of ChIP-Seq data (from the GEO database) around the SOCS5 gene revealed a STAT3 binding site located in the SOCS5 promoter (chr2:46925605-46925927), suggesting a direct link between STAT3 activation and SOCS5 expression. To determine whether IL-10-induced STAT3 activation was sufficient to upregulate SOCS5, we treated monocytes from normal donors with IL-10 and monitored SOCS5 protein levels over time. IL-10 treatment induced an increase in SOCS5 that was maintained up to 120h (Figure 5C and 5D). As SOCS5 expression increased, the activating tyrosine phosphorylation of STAT3 and STAT6 (following IL-4 stimulation), which are negatively regulated by SOCS5 [24, 25, 27], steadily decreased (Figure 5C and 5D).

Since IL-4Rα is a STAT3 target gene [34], we considered the possibility that elevated IL-10 levels in CLL patients could lead to enhanced expression of IL-4Rα on monocytes. IL-10 treatment *in vitro* significantly increased mRNA expression of IL-4Rα in normal monocytes (Figure 5E), suggesting that IL-10-induced STAT3 phosphorylation could explain the elevated levels of IL-4Rα observed in monocytes from CLL patients. We next determined whether IL-10 signaling could mimic the phenotype observed in Mo-DCs from CLL. Monocytes from healthy donors were differentiated into DCs in the presence or absence of IL-10 and evaluated for the expression of CD14, HLA-DR, CD11c, CD80, CD86, CD83 and CD40. The expression of these surface molecules in Mo-DCs differentiated with IL-10 was similar to the pattern observed in Mo-DCs from CLL patients (Figure 5F). These findings suggest that IL-10-induced STAT3 phosphorylation increases SOCS5 expression in monocytes in CLL patients, which then negatively regulates IL-4R signaling through inhibiting tyrosine phosphorylation of STAT6.

### Overexpression of SOCS5 in healthy monocytes impairs DCs differentiation

**Figure 6:**
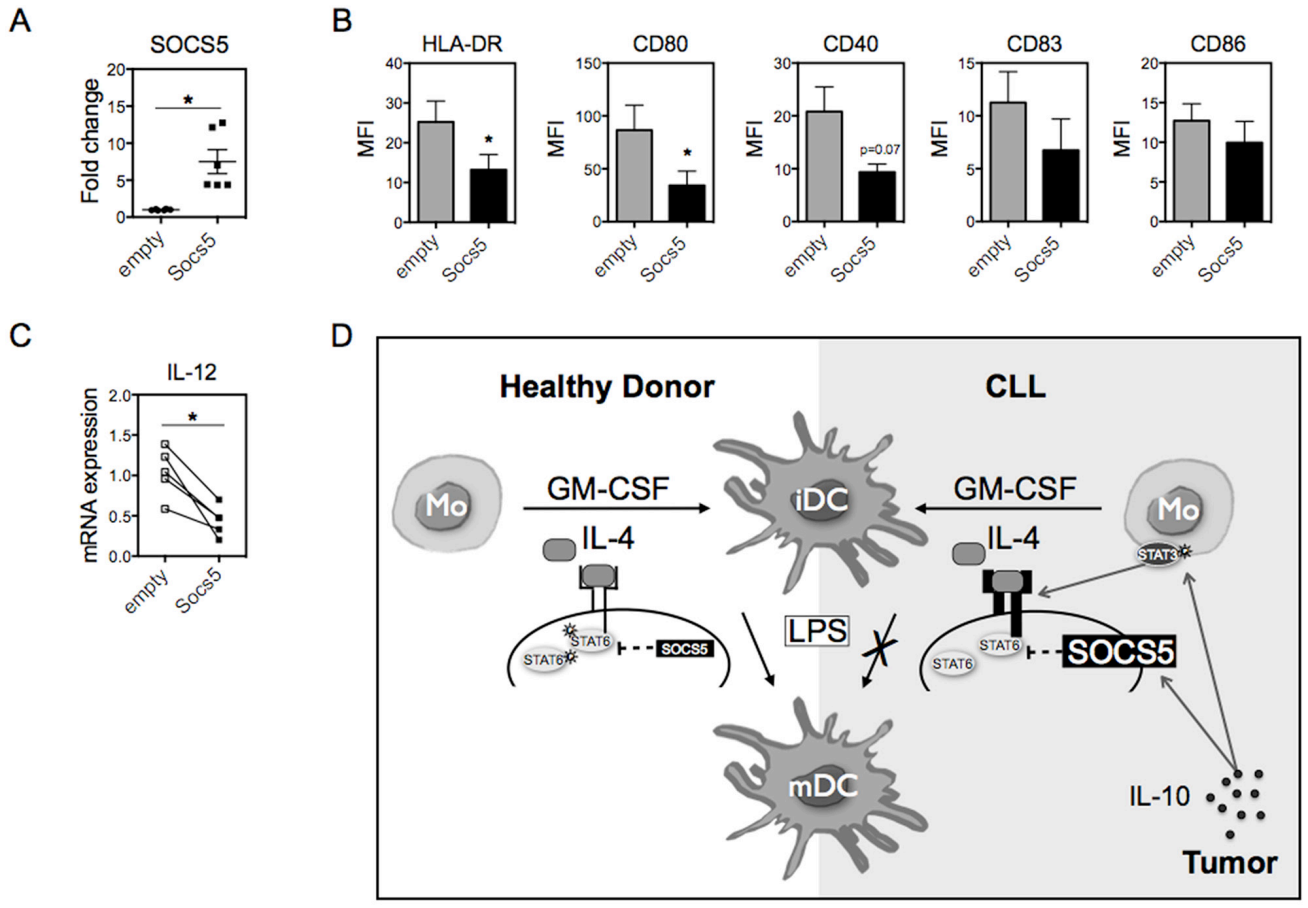
SOCS5 overexpression impairs Mo-DC differentiation **A.** Monocytes from HD were transduced with a lentivirus expressing SOCS5 or empty vector for 4 hours. mRNA expression of SOCS5 24h after transduction was measured by qRT-PCR (normalized to 18S RNA) and shown as fold change relative to empty vector. **B.** Transduced monocytes were differentiated into DCs with IL-4 and GM-CSF, and on day 5 were stimulated with LPS for 24h. The expression of the indicated surface molecules were assessed by flow cytometry. N=5. **C.** mRNA for IL-12 was quantitated Mo-mDCs after transduction with SOCS5 or empty vector. N=5. Data are shown as mean ± SEM. Unpaired t-test. *p<0.05. **D.** Schematic representing the molecular difference in DC differentiation and maturation between healthy individuals and CLL patients. In healthy individuals, IL-4 induces the activation of the downstream transcription factor STAT6, and full maturation of Mo-DCs is achieved following LPS stimulus. In CLL patients, the presence of IL-10 (likely produced by the leukemia cells) increases the phosphorylation of STAT3 in monocytes, resulting in enhanced expression of both the IL-4 receptor and SOCS5. The elevated expression of SOCS5 inhibits maximal activation of STAT6, thereby impairing the phenotypic maturation of Mo-DCs induced by LPS.

To determine the role of SOCS5 in Mo-DCs, we first attempted to use RNA interference to deplete SOCS5 in monocytes from CLL patients, though low viability of these cells following use of RNA interference precluded these experiments. Thus, we chose to overexpress SOCS5 in monocytes derived from healthy volunteers to directly determine whether SOCS5 could impair Mo-DC differentiation. We first exogenously expressed SOCS5 by transducing monocytes isolated from healthy donors with lentiviral vectors expressing SOCS5 and GFP or GFP alone. The efficiency of transduction was monitored by qRT-PCR for SOCS5 mRNA expression (Figure 6A). These monocytes were then differentiated to DCs by the addition of IL-4 and GM-CSF, and were induced to mature with LPS. SOCS5-transduced Mo-DCs showed lower expression of HLA-DR, CD80 and CD40 than Mo-DCs transduced with empty vector (Figure 6B). Similarly, the levels of the pro-inflammatory cytokine IL-12 were notably decreased in SOCS5-transduced Mo-DCs (Figure 6C). These data confirm that enhanced expression of SOCS5 in the precursor of DCs is sufficient to impair DC differentiation (Figure 6D).

## DISCUSSION

Severe recurrent infections are the major life-threatening complication associated with the immunodeficiency in CLL and are responsible for high rates of non-relapse mortality [35, 36]. Functional impairment of DCs not only contributes to an increased infection rate but it also may allow for tumor escape from immune control. Here, we focused on signal transduction pathways that regulate the expression of genes necessary for the immune response and found elevated SOCS5 as a potential mechanism for the impaired DC function seen in CLL patients. Monocytes from CLL patients exhibit high IL-4R expression, likely due to the effects of elevated circulating levels of IL-10 in CLL patients [15, 23, 33]. However, activation of the downstream transcription factor STAT6 is inhibited due to increased expression of the negative regulator SOCS5 (which is also likely a result of elevated IL-10). This decoupling of IL-4 from STAT6 activation impairs the phenotypic and functional maturation of DCs from CLL patients (Figure 6D).

Monocytes derived from CLL patients were unable to fully differentiate into DCs, generating functionally defective cells that resemble immature myeloid dendritic cells. LPS stimulation did not enhance the expression of the costimulatory molecules HLA-DR and CD40 or the activation marker CD83 on the surface of CLL Mo-DCs, nor did it enhance production of IL-12, to levels comparable to those in healthy donors, perhaps due to lower expression of the LPS receptor complex. The upregulation of these surface molecules and production of pro-inflammatory cytokines are essential for phenotypic and functional maturation of DCs [37, 38]. By contrast, CLL Mo-DCs produced immunosuppressive factors, such as IL-10 and TGF-β. Similarly, Mo-DCs derived from breast cancer patients were reported as less sensitive to maturation-inducing agents than DCs obtained from healthy donors [39]. The phenotypic pattern we identified in DCs differentiated *in vitro* from CLL monocytes was very similar to the defective phenotype described in circulating DCs from CLL patients [15].

CLL Mo-DCs co-cultured with T cells showed poor allo-stimulatory capacity and induced low secretion of IFN-γand TNF in the co-culture assay, as previously reported [5]. These findings indicate that the phenotypic dysfunction found in CLL Mo-DCs impairs their ability to induce Th1 and inflammatory CD8 T-cells. Nevertheless, CLL Mo-DCs induced higher frequency of Tregs (CD4^+^CD127^low^CD25^hi^FOXP3^+^), which has been seen in other cancer types [40]. These findings are consistent with data showing increased frequency of Tregs in the peripheral blood of CLL patients, which is related to advanced stage of disease [7, 30, 41–43].

Although it has been suggested that the compromised differentiation of DCs in cancer could result from an alteration in monocytes, the mechanism underlying this defect has been uncertain. We characterized monocytes from patients with CLL to explore this hypothesis. Although no difference was observed in the expression of monocyte surface markers, monocytes from CLL patients had abnormal signaling through the IL-4 receptor. The differentiation of DCs to a functionally competent cell requires IL-4R signaling and the activation of the downstream transcription factor STAT6 [20, 31]. Monocytes from CLL patients displayed higher levels of IL-4Rα (CD124) than healthy donors, but, paradoxically, IL-4-induced STAT6 activation was decreased. Reduced STAT6 activation in both human and mouse models maintains DCs in an immature state, resembling tolerogenic cells [21, 31]. Thus, alteration of STAT6 signaling and the low expression of receptors, such as TLRs, that trigger Mo-DC maturation in CLL prevent full differentiation of Mo-DCs.

A role for SOCS5 in DC differentiation or function has not previously been described. We have shown that elevated SOCS5 expression can attenuate the effects of IL-4 in driving the differentiation of monocytes into functional dendritic cells. The binding of SOCS5 to IL-4Rα prevents its interaction with Jak family kinases, blocking IL-4-dependent STAT6 activation [26]. Importantly, STAT3 is a convergence point for a large number of suppressive factors released by malignant cells, including IL-10, which is known to be produced by CLL cells [15, 23, 33]. Treatment of monocytes from healthy donors with IL-10, which activates STAT3, led to similar signaling events as that observed in patients, including enhanced expression of SOCS5 and inhibition of STAT6 activation, resulting in defective Mo-DC differentiation analogous to that seen in CLL-derived Mo-DCs. The ectopic expression of SOCS5 in monocytes from healthy donors was sufficient to inhibit the expression of several key proteins in Mo-DCs, including HLA-DR, CD80, and CD40, and to suppress the production of IL-12. Together, our data indicate that SOCS5 overexpression negatively affects dendritic cell function.

The discovery of new therapeutic targets that restore DC function is important not only to induce immunity against cancer and infections, but also to improve the efficacy of other therapeutic approaches. In particular, immunotherapeutic approaches that involve enhanced tumor antigen presentation by DCs would also benefit from this strategy [3, 44–46]. While SOCS5 may not be the only factor mediating DC dysfunction in cancer, it clearly contributes to DC defects in CLL patients. Our data show that tumor-released soluble factors are sufficient to induce STAT3 activation, leading to increased expression of IL-4Rα in monocytes (Figure 6D). However, IL-4R signaling is decoupled from activation of its downstream mediator STAT6 by enhanced expression of SOCS5, thereby preventing the differentiation of functionally mature DCs. These findings provide new insight into the molecular regulation of DC differentiation and suggest that SOCS5 is a potential therapeutic target for reversing cancer-associated immune suppression.

## MATERIALS AND METHODS

### Subjects

Peripheral blood samples were collected from healthy donors or CLL patients with informed consent in accordance with the Declaration of Helsinki, under a Dana-Farber Cancer Institute Institutional Review Board-approved protocol. All patients had active disease and were either previously untreated or were refractory to therapy. The white blood cell (WBC) count ranged from 45 to 445 x10^3^/mm^3^ (mean 116 x10^3^/mm^3^) and lymphocytes accounted for 63-94% (mean 82%) of WBCs.

### Generation of Mo-DCs

PBMCs were isolated by Ficoll-Paque density gradient centrifugation. CD14^+^ monocytes were obtained from PBMCs by positive selection using microbeads according to the manufacturer's protocol(Miltenyi Biotec). The purity of monocytes from healthy donors was greater than 90%, and the purity from CLL patients was greater than 85%. Although monocytes can be further subdivided based on relative expression of CD14 and CD16, given that approximately 90% display the classical phenotype (CD14^+^CD16^−^), and that monocytes represent only approximately 1% of PBMC in CLL patients (limiting the ability to analyze subtypes), we chose to use all CD14^+^ cells for these experiments. To generate DCs, CD14^+^ monocytes were cultured in RPMI 1640 complete medium (10% heat inactivated fetal bovine serum, 1% GlutaMAX, 1mM sodium pyruvate, 0.5% MEM-amino acids, 1% MEM-Vitamin, 0.07 mM β-ME, 1% penicillin/streptomycin; Gibco^®^, Grand Island, NY, USA) supplemented with GM-CSF (50 ng/ml; PeproTech, Rocky Hill, NJ, USA) and IL-4 (50 ng/ml; PeproTech). After 5 days, immature Mo-DCs (Mo-iDCs) were induced to mature with LPS (*Escherichia coli;* 100ng/mL; Sigma-Aldrich, St. Louis, MO, USA). At day 6, mature Mo-DCs (Mo-mDCs) were harvested for further experiments.

### T cell proliferation assay

Mature Mo-DCs from healthy donors or CLL patients were harvested at day 6 of culture. These cells were then cocultured with bead-purified allogeneic CD3^+^ T cells labeled with CellTrace Violet (Molecular Probes®). Cells were cultured at 37°C in 5% CO_2_ for 5 days. Cell proliferation was quantified by flow cytometry. The proliferation index was calculated withFlowJo 8.7 software (Ashland, OR).

### Flow cytometric analysis

For surface staining, cells were incubated with antibodies diluted in PBS containing 2% FBS for 20 min, on ice. Conjugated antibodies to CD11c (FITC), CD124 (PE), CD14 (PERCP), CD80 (PE-Cy7), CD83 (APC), CD40 (APC-Cy7), CD86 (pacific blue) and HLA-DR (V500) were from BD Biosciences. Conjugated antibodies to CD4 (APC, APC-Cy7 and Alexa-Fluor 488), CD8 (PE and PERCP), CD25 (APC-Cy7) and CD127 (PE-Cy7) were from Biolegend. Intracellular cytokine staining was performed with BD Cytofix/Cytoperm kit (BD Biosciences). Cells were stimulated with Leukocyte Activation Cocktail with GoldiPlug (BD Biosciences) for 5 hours at 37°C in 5% CO_2_, and were then stained with PE anti-IL-4 and APC anti-IFNγ (Biolegend). Regulatory T cells (CD4^+^CD127^−^CD25^+^Foxp3^+^) were stained for intracellularFoxp3 using the APC anti-human Foxp3 antibody kit (eBioscience). Flow cytometry was performed using a FACSCanto II (Becton Dickinson, Franklin Lakes, NJ), and theresults were analyzed with FlowJo 8.7 software.

### Intracellular staining of phosphorylated STAT6 and STAT3

Monocytes from CLL patients or healthy donors were left unstimulated or stimulated for 15 min at 37°C with recombinant human IL-4 (50ng/mL) to stimulate STAT6 phosphorylation, or with recombinant human IL-6 (50ng/mL) to stimulate STAT3 phosphorylation. Intracellular staining of phosphorylated STAT6 or STAT3 was performed according to the BD Bioscience protocol. Samples were acquired on a BD FACSCanto II instrument and analyzed with FlowJo software.

### Determination of cytokine production

Supernatant from Mo-mDC culture or from co-culture assays with CD3^+^ T cells was harvested, and quantitation was performed for human IL-12p70, TNF, IL-10 and IFN-γ using ELISA MAX™ Deluxe (Biolegend®, San Diego, CA, USA). Analysis was performed using SpectraMax M3 (Molecular Devices).

### IL-10 treatment of monocytes

Monocytes from healthy donors were isolated as described above and incubated with IL-10 (50 ng/ml; PeproTech) for 24, 48, 72, 96 or 120h. Cells were then harvested for further experiments. To determine the effect of IL-10 on Mo-DC generation, IL-10 (50 ng/ml) was added to monocyte cultures in combination with IL-4 (50 ng/ml) and GM-CSF (50 ng/ml). On the fifth day of culture, cells were stimulated with LPS (100 ng/mL) for 24 hours and analyzed by flow cytometry.

### Ectopic expression of SOCS5 in monocytes

The human cDNA ORF clone of SOCS5, cloned in pLenti-C-mGFP vector, was obtained from Origen Technologies (Rockville, MD, USA) and lentivirus was produced as described [18, 47]. Monocytes were infected with lentivirus expressing SOCS5 or empty vector 24 hours after cell isolation in the presence of 4 μg/mL polybrene (Sigma-Aldrich). Infected cells were centrifuged at 2500 rpm, 32°C for 1h30min. Medium was changed 4 hours after transduction and monocytes were incubated overnight. Then, transduced monocytes were harvested or differentiated into DCs by adding IL-4 (50 ng/ml) and GM-CSF (50 ng/ml). On the fifth day following cytokine addition, cells were stimulated with LPS (100 ng/mL). After 24 hours, mature Mo-DCs were analyzed by flow cytometry or harvested for RNA analysis. The transduction efficiency was approximately 80%.

### RNA isolation and quantitative RT-PCR (qRT-PCR)

RNA was harvested using the RNeasy Mini Kit from Qiagen (Valencia, CA). cDNA was synthesized using the TaqMan Reverse Transcription kit (Applied Biosystems, Foster City, CA). qPCR was performed in triplicate using SYBR select master mix (Applied Biosystems) on ABI Prism 7500 Sequence Detection System (Applied Biosystems). RNA expression was normalized to 18S RNA, and the fold change was determined by normalizing the expression in each sample to the mean values of control cells from healthy donors. Data are expressed as mean fold increase ± standard error ofthe mean (SEM). Primer sequences are provided in Supplementary Table SI.

### Immunoblotting

Whole cell extracts were prepared using lysis buffer (50 mM Tris, pH 8.0, 250 mM NaCl, 0.5% NP-40) containing protease and phosphatase inhibitors. Protein lysates were resolved by 8% SDS-PAGE and immunoblotted with primary antibodies specific for phosphorylated STAT6 and phosphorylated STAT3 (Cell Signaling, Boston, MA); calnexin (Santa Cruz Biotechnology, Santa Cruz, CA); and SOCS5 (Abcam). Band intensity was quantitated using Image J software (NIH).

### Statistical analysis

Data are shown as means ± standard error of the mean (SEM). Two group comparisons were carried out using two-tailed unpaired Student t test; otherwise, one-way analysis of variance (ANOVA) followed by Tukey post test for multiple comparisons was performed. Analyses were conducted using GraphPad PRISM 6 software, and differences were considered significant at p < 0.05.

## SUPPLEMENTARY FIGURES AND TABLE


