# Supplementary material for: Deregulation of SOCS5 suppresses dendritic cell function in chronic lymphocytic leukemia
Source: Oncotarget. 2016 Jun 15;7(29):46301–14. doi: 10.18632/oncotarget.10093 (PMC5216799; doi:10.18632/oncotarget.10093)
Supplement: Supplementary file 1 [file oncotarget-07-46301-s001.pdf]

Deregulation of SOCS5 suppresses dendritic cell function in chronic lymphocytic leukemia

SUPPLEMENTARY FIGURES AND TABLE

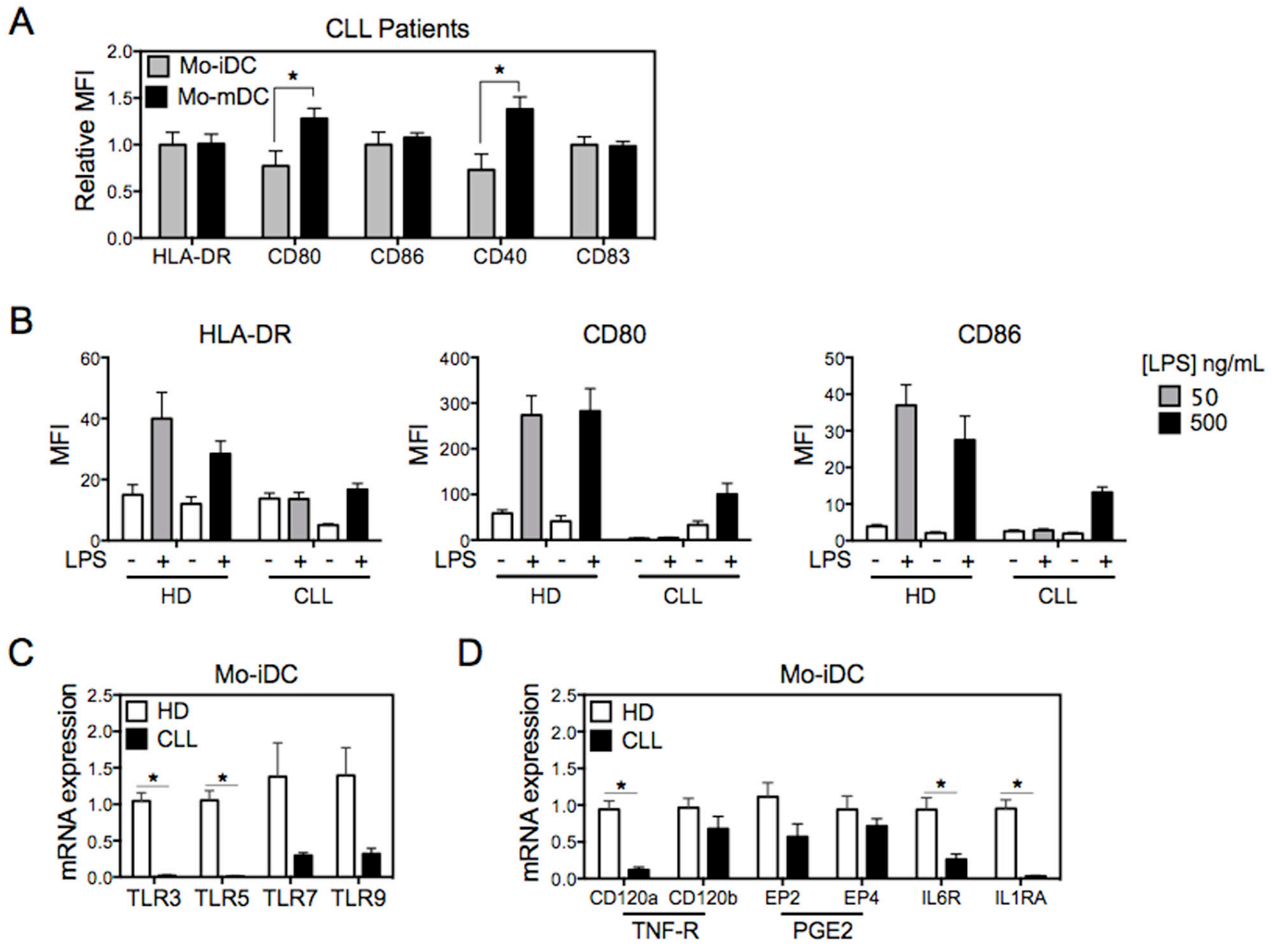

Supplementary Figure S1: Mo-DCs from CLL patients are less responsive to LPS stimulation. A. Expression of the indicated Mo-DC surface molecules was analyzed by flow cytomctry in unstimulated (Mo-iDC) and LPS-stimulated (Mo-mDC) cells derived from CLL patients. Doublets were excluded from analysis and Mo-DCs were defined as CDl4^{-}HLA-DR^{+}. N=5. **B.** Mo-DCs were differentiated from HD and CLL, and on the 5th day cells were left unstimulated or were stimulated with the indicated concentration of LPS. N=4. **C.** and **D.** mRNA expression of TLRs (C) and receptors for TNF, PEG2, IL-6 and IL-1β (D) was analyzed in MoiDCs by qRT-PCR, nonnalized to 18S RNA. N=6. Data are shown as mean ± SEM. Unpaired t-test. *p<0.05 .

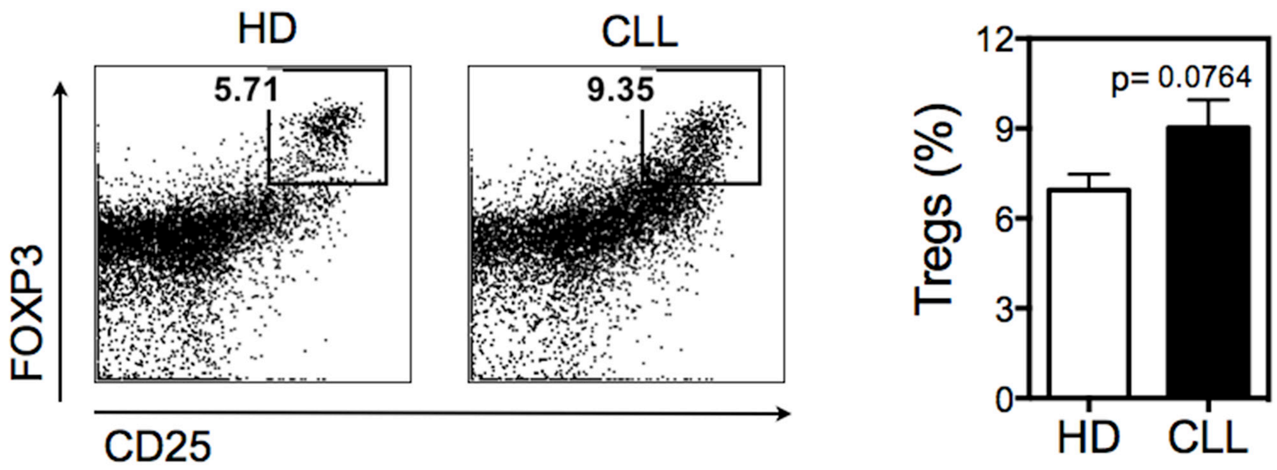

Supplementary Figure S2: Mo-DC from CLL induces and expands a higbee frequency of regulatory T cells. Mo-mDCs from HD and CLL were co-cultured with bead-purified allogeneic CD3 T-cel1s for 5 days. The frequency of Tregs was evaluated, and shown as representative dot plots (left panel) and bar graphs (right panel). Tregs were defined as $CD4^{+}CD127^{lo}CD25^{hi}FOXP3^{+}$. N=3 run in duplicate. Data are shown as mean ± SEM. Unpaired t-tcst. *$p<0.05$.

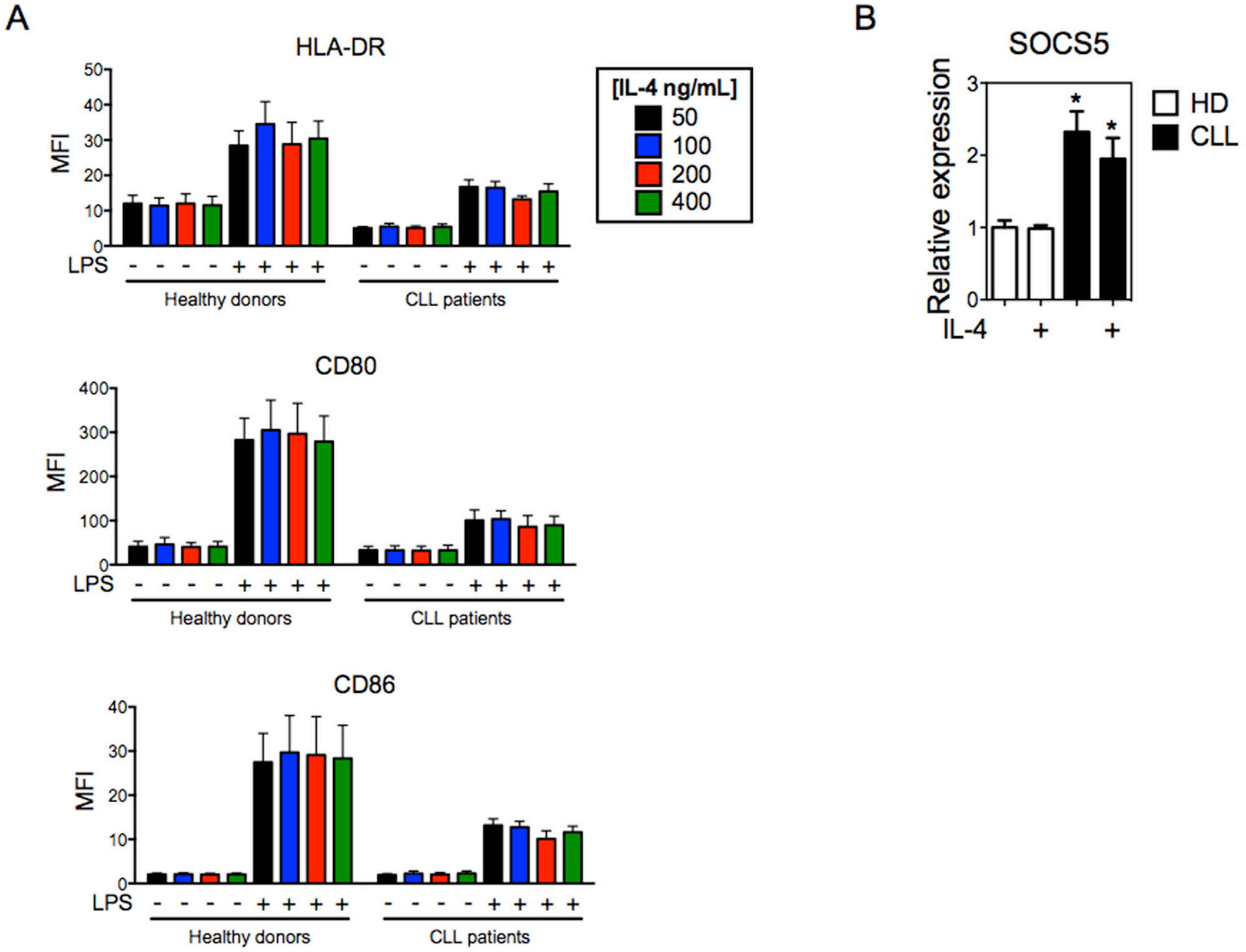

Supplementary Figure S3: Effects of IL-4 on cell surface molecule and SOCS5 expression. A. Mo-DCs were differentiated from HD and CLL patients in the presence of increasing concentrations of IL-4, as indicated. On the 5th day of culture, cells were stimulated with LPS and evaluated by flow cytomctry for the expression of HLA-DR and costimulatory molecules. Mo-DCs were defined as CD14^{-} HLA-DR^{+}, excluded of doublets. N=4. **B.** mRNA expression of SOCS5 in monocytcs from HD and CLL patients unstimulated or stimulated with IL-4 for 30 min was analyzed by qRT-PCR (normalized to 18S RNA). N=6. One-way ANOVA Tukey mUltiple comparison test. *p<0.05, compared with HD. Data are shown as mean ± SEM.

Supplemental Table SI: Sequences of primers used in qRT-PCR assays

Genes	Forward	Reverse
SOCS1	TGGTGCACACAACCAGGTG	GAGGAGGAGGAAGAGGAGGA
SOCS2	CTTGAGCCCTCCGGGAAT	TCTCTTTGGCTTCATTAACAGTCA
SOCS3	TCAAGACCTTCAGCTCCAAG	TGACGCTGAGCGTGAAGAAG
SOCS5	ACCCAGAGTTCATTGGATGC	CCCACAGTATCCTGCAACCT
STAT6	TCCCAGAGCACTTCCTCTTC	GTCCAGGACACCATCAAACC
STAT1	TACTCCAGGCCAAAGGAAGC	AGAAGGGTGAACTTCAGACACA
PTPRC	AGCACCTACCCTGCTCAGAA	TTCAGCCTGTTCCTTTGCTT
CDK6	TCTTGCTCCAGTCCAGCTAC	TCTCCTGGGAGTCCAATCAC
TNFSF10	ATGGCTATGATGGAGGTCCAG	TTGTTCTGCATCTGCTTCAGC
TRIM22	GGATGCCAGCAGGCTCATCTCAG	TTCAGCATCACGTCCACCCAGTAGT
IL-12p40	TCCTGCCCAGAGCAAGATGTGT	ATGGCCACGAGGGGAGATGC
IL-6	CTCGAGCCCACCGGGAACGA	GGACCGAAGGCGCTTGTGGA
IL-10	CCTGGAGGAGGTGATGCCCCA	ATCGATGACAGCGCCGTAGCC
TNF	CGGCCCCCAGAGGGAAGAGT	GAGGGTTTGCTACAACATGGGCT
TGF-β1	GGAAATTGAGGGCTTTCGCC	CCGGTAGTGAACCCGTTGAT
TLR4	GGATGATGCCAGGATGATGTCT	ATTAGGAACCACCTCCACGC
MD2	GCACATTTTCTACATTCCAAGGAG	TGCGCTTTGGAAGATTCATGG
MyD88	AGAGGTTGGCTAGAAGGCCA	CTTCTGATGGGCACCTGGA
TRIF	CCGGATCCCTGATCTGCTTGG	GGTGAAGGCATGTTCCACACT
CD120b	TGTGACAGCCTTGGGTCTAC	CAAGTGAGGCACCTTGGCTT
CD120a	CTAGACACTGATGACCCCGC	GAATTCCTTCCAGCGCAACG
IL-1R	TGGGGAAGACATTGTTGAGGT	TTCAGATGAACCACCCAGCC
EP2	GCTCCTTGCCTTTCACGATTT	AGGATGGCAAAGACCCAAGG
EP4	CCGCTCGTGGTGCGAGTATT	GGATGGGGTTCACAGAAGCA
IL-6R	CCACCCCCATGCAGGCACTTAC	GAACCTGGGAGGCTTGTCGCAT
IL-4Rα	AACGACCCGGCAGATTTCAG	CGTGCCCTGTAGGAAATCCC
18sRNA	GTAACCCGTTGAACCCCATT	CCATCCAATCGGTAGTAGCG
